# Food-related illness and death in the United States.

**DOI:** 10.3201/eid0505.990502

**Published:** 1999

**Authors:** P S Mead, L Slutsker, V Dietz, L F McCaig, J S Bresee, C Shapiro, P M Griffin, R V Tauxe

**Affiliations:** Division of Bacterial and Mycotic Diseases, Centers for Disease Control and Prevention, Atlanta, Georgia 30333, USA. pfm0@cdc.gov

## Abstract

To better quantify the impact of foodborne diseases on health in the United States, we compiled and analyzed information from multiple surveillance systems and other sources. We estimate that foodborne diseases cause approximately 76 million illnesses, 325,000 hospitalizations, and 5,000 deaths in the United States each year. Known pathogens account for an estimated 14 million illnesses, 60, 000 hospitalizations, and 1,800 deaths. Three pathogens, Salmonella, Listeria, and Toxoplasma, are responsible for 1,500 deaths each year, more than 75% of those caused by known pathogens, while unknown agents account for the remaining 62 million illnesses, 265,000 hospitalizations, and 3,200 deaths. Overall, foodborne diseases appear to cause more illnesses but fewer deaths than previously estimated.


### Food-Related Illness and Death in the United States

**Paul S. Mead, Laurence Slutsker, Vance Dietz, Linda F. McCaig, Joseph S. Bresee, Craig Shapiro, Patricia M. Griffin, and Robert V. Tauxe**
Centers for Disease Control and Prevention, Atlanta, Georgia, USA

To better quantify the impact of foodborne diseases on health in the United States, we compiled and analyzed information from multiple surveillance systems and other sources. We estimate that foodborne diseases cause approximately 76 million illnesses, 325,000 hospitalizations, and 5,000 deaths in the United States each year. Known pathogens account for an estimated 14 million illnesses, 60,000 hospitalizations, and 1,800 deaths. Three pathogens, *Salmonella, Listeria*, and *Toxoplasma*, are responsible for 1,500 deaths each year, more than 75% of those caused by known pathogens, while unknown agents account for the remaining 62 million illnesses, 265,000 hospitalizations, and 3,200 deaths. Overall, foodborne diseases appear to cause more illnesses but fewer deaths than previously estimated.

More than 200 known diseases are transmitted through food (1). The causes of foodborne illness include viruses, bacteria, parasites, toxins, metals, and prions, and the symptoms of foodborne illness range from mild gastroenteritis to life-threatening neurologic, hepatic, and renal syndromes. In the United States, foodborne diseases have been estimated to cause 6 million to 81 million illnesses and up to 9,000 deaths each year (2-5). However, ongoing changes in the food supply, the identification of new foodborne diseases, and the availability of new surveillance data have made these figures obsolete. New, more accurate estimates are needed to guide prevention efforts and assess the effectiveness of food safety regulations.

Surveillance of foodborne illness is complicated by several factors. The first is underreporting. Although foodborne illnesses can be severe or even fatal, milder cases are often not detected through routine surveillance. Second, many pathogens transmitted through food are also spread through water or from person to person, thus obscuring the role of foodborne transmission. Finally, some proportion of foodborne illness is caused by pathogens or agents that have not yet been identified and thus cannot be diagnosed. The importance of this final factor cannot be overstated. Many of the pathogens of greatest concern today (e.g., *Campylobacter jejuni*, *Escherichia coli* O157:H7, *Listeria monocytogenes, Cyclospora cayetanensis*) were not recognized as causes of foodborne illness just 20 years ago.

In this article, we report new estimates of illnesses, hospitalizations, and deaths due to foodborne diseases in the United States. To ensure their validity, these estimates have been derived by using data from multiple sources, including the newly established Foodborne Diseases Active Surveillance Network (FoodNet). The figures presented include estimates for specific known pathogens, as well as overall estimates for all causes of foodborne illness, known, unknown, infectious, and noninfectious.

### Data Sources

Data sources for this analysis include the Foodborne Diseases Active Surveillance Network (FoodNet) (6), the National Notifiable Disease Surveillance System (7), the Public Health Laboratory Information System (8), the Gulf Coast States Vibrio Surveillance System (9), the Foodborne Disease Outbreak Surveillance System (10), the National Ambulatory Medical Care Survey (11), the National Hospital Ambulatory Medical Care Survey (12-14), the

Address for correspondence: Paul S. Mead, Division of Bacterial and Mycotic Diseases, Centers for Disease Control and Prevention, Mail Stop A38, 1600 Clifton Road, Atlanta, GA 30333, USA; fax: 404-639-2205; e-mail: pfm0@cdc.gov.

National Hospital Discharge Survey (15), the National Vital Statistics System (16), and selected published studies.

Established in 1996, FoodNet is a collaborative effort by the Centers for Disease Control and Prevention, the U.S. Department of Agriculture, the U.S. Food and Drug Administration, and selected state health departments. FoodNet conducts active surveillance for seven bacterial and two parasitic foodborne diseases within a defined population of 20.5 million Americans (6). Additional surveys conducted within the FoodNet catchment area provide information on the frequency of diarrhea in the general population, the proportion of ill persons seeking care, and the frequency of stool culturing by physicians and laboratories for selected foodborne pathogens.

The National Notifiable Disease Surveillance System (7) and the Public Health Laboratory Information System (8) collect passive national surveillance data for a wide range of diseases reported by physicians and laboratories. The Gulf Coast States Vibrio Surveillance System collects reports of *Vibrio* infections from selected states (9), and the Foodborne Disease Outbreak Surveillance System receives data from all states on recognized foodborne illness outbreaks (defined as two or more cases of a similar illness resulting from ingestion of a common food) (10).

As components of the National Health Care Survey, the National Ambulatory Medical Care Survey and the National Hospital Ambulatory Medical Care Survey measure health care use in various clinical settings, including physician offices and hospital emergency and outpatient departments (11-14). These surveys collect information on patient characteristics, patient symptoms or reasons for visit, provider diagnosis, and whether the patient was hospitalized. Up to three symptoms are recorded using a standard classification (17), and up to three provider diagnoses are recorded according to the International Classification of Diseases, 9th Revision, Clinical Modifications (ICD-9-CM,18) (Table 1).

The National Hospital Discharge Survey, another component of the National Health Care Survey, is a representative annual sample of discharge records from approximately 475 nonfederal short-stay hospitals (15). The information collected includes up to seven principal discharge diagnoses classified by ICD-9-CM codes (18). Because these data include information on condition at discharge, they can be used as a source of information on in-hospital deaths. Additional information on food-related deaths was obtained from the National Vital Statistics System, which collects death certificate data on causes of death classified by 3- or 4-digit ICD-9 codes (16).

In addition to information from these formal surveillance systems, we used data from two published population-based studies. The Tecumseh study was conducted from 1965 through 1971 in 850 households in Tecumseh, Michigan, with an emphasis on households with young children (19). Households were telephoned weekly to identify incident cases of self-defined diarrhea, vomiting, nausea, or stomach upset. The Cleveland study was conducted among a selected group of 86 families followed from 1948 through 1957 (20). A family member

Table 1. ICD-9-CM codes and associated conditions

| Code | Condition |
|---|---|
| 001 | Cholera |
| 002 | Typhoid fever |
| 003 | *Salmonella* |
| 004 | Shigellosis |
| 005.0 | Staphyloccocal food poisoning |
| 005.1 | Botulism |
| 005.2-005.3 | Other *Clostridia* |
| 005.4 | *Vibrio parahaemolyticus* |
| 005.8-005.9 | Other and unspecified bacterial food poisoning |
| 006 | Amebiasis |
| 007.1 | Giardiasis |
| 007.0, 007.2-007.9 | Other protozoal intestinal infections |
| 008.00, 008.09 | Misc. *Escherichia coli* |
| 008.01 | Enteropathogenic *E. coli* |
| 008.02 | Enterotoxigenic *E. coli* |
| 008.03 | Enteroinvasive *E. coli* |
| 008.04 | Enterohemorrhagic *E. coli* |
| 008.43 | *Campylobacter* |
| 008.44 | *Yersinia* |
| 008.41-2, 008.46-9, 008.5 | Misc. bacterial |
| 008.61 | Rotavirus |
| 008.62 | Adenovirus |
| 008.63 | Norwalk virus |
| 008.64 | Other small round structured viruses |
| 008.65 | Calicivirus |
| 008.66 | Astrovirus |
| 008.67 | Enterovirus |
| 008.69, 008.8 | Other virus |
| 009. | Ill-defined intestinal infections |
| 558.9 | Other noninfectious gastroenteritis |

recorded occurrences of gastrointestinal illnesses and associated symptoms on a monthly tally sheet. Both studies also collected information on extraintestinal illnesses (e.g., respiratory illness). Other studies with similar designs were not included in our analysis, either because they were relatively small or because they did not provide information on the desired endpoints.

### The Study

### Food-Related Illness and Death from Known Pathogens

#### Total Cases

To estimate the total number of foodborne illnesses caused by known pathogens, we determined the number of reported cases for each pathogen, adjusted the figures to account for underreporting, and estimated the proportion of illnesses specifically attributable to foodborne transmission. Although data from various periods were used, adjustments for changes in population size had minimal effect on the final estimates and were therefore omitted.

Cases may be reported in association with documented foodborne outbreaks, through passive surveillance systems (e.g., the National Notifiable Disease Surveillance System, the Public Health Laboratory Information System), or through active surveillance systems (e.g., FoodNet). Sporadic illness caused by some pathogens (e.g., *Bacillus cereus*, *Clostridium perfringens*, *Staphylococcus aureus*) is not reportable through passive or active systems; hence, the only cases reported are those related to outbreaks. For these pathogens, we have assumed that if diagnosed sporadic cases were reported, the total number would be 10 times the number of outbreak-related cases. This multiplier is based on experience with pathogens for which data are available on both sporadic and outbreak-associated cases (e.g., reported cases of *Salmonella* or *Shigella*, Table 2). For all pathogens, the number of outbreak-related cases was calculated as the average annual number of such cases reported to CDC from 1983 to 1992, the most recent years for which published outbreak data are available. For pathogens also under passive surveillance, we used the average number of cases reported to CDC from 1992 through 1997, and for pathogens under active surveillance through FoodNet, we used the average rate observed for the surveillance population from 1996 to 1997 and applied this to the total 1997 U.S. population (with some modification for *E. coli* O157:H7; Appendix).

Irrespective of the surveillance system, many cases of foodborne illness are not reported because the ill person does not seek medical care, the health-care provider does not obtain a specimen for diagnosis, the laboratory does not perform the necessary diagnostic test, or the illness or laboratory findings are not communicated to public health officials. Therefore, to calculate the total number of illnesses caused by each pathogen, it is necessary to account for underreporting, i.e., the difference between the number of reported cases and the number of cases that actually occur in the community. For *Salmonella*, a pathogen that typically causes nonbloody diarrhea, the degree of underreporting has been estimated at ~38 fold (Voetsch, manuscript in preparation) (21). For *E. coli* O157:H7, a pathogen that typically causes bloody diarrhea, the degree of underreporting has been estimated at ~20 fold (22). Because similar information is not available for most other pathogens, we used a factor of 38 for pathogens that cause primarily nonbloody diarrhea (e.g., *Salmonella, Campylobacter*) and 20 for pathogens that cause bloody diarrhea (e.g., *E. coli* O157:H7, *Shigella*). For pathogens that typically cause severe illness (i.e., *Clostridium botulinum, Listeria monocytogenes*), we arbitrarily used a far lower multiplier of 2, on the assumption that most cases come to medical attention. Details of the calculations for each specific pathogen and rationale are provided in the Appendix. Where information from both active and passive reporting was available, we used the figure from active surveillance when estimating the total number of cases.

Having estimated the number of cases caused by each pathogen, the final step was to estimate for each the percentage of illness attributable to foodborne transmission. The total number of cases was then multiplied by this percentage to derive the total number of illnesses attributable to foodborne transmission. The rationale for each estimate is presented in the Appendix; although precise percentages are generally difficult to justify, in most instances there is ample support for the approximate value used.

Results are presented in Tables 2 and 3. Known pathogens account for an estimated 38.6

Table 2. Reported and estimated[a] illnesses, frequency of foodborne transmission, and hospitalization and case-fatality rates for known foodborne pathogens, United States

| Disease or Agent | Estimated total cases | Reported Cases by Surveillance Type: Active | Passive | Outbreak | % Foodborne transmission | Hospital-ization rate | Case-fatality rate |
|---|---|---|---|---|---|---|---|
| **Bacterial** | | | | | | | |
| *Bacillus cereus* | *27,360* | | *720* | 72 | *100* | 0.006 | 0.0000 |
| Botulism, foodborne | *58* | | 29 | | *100* | 0.800 | 0.0769 |
| *Brucella* spp. | *1,554* | | 111 | | *50* | 0.550 | 0.0500 |
| *Campylobacter* spp | *2,453,926* | *64,577* | 37,496 | 146 | *80* | 0.102 | 0.0010 |
| *Clostridium perfringens* | *248,520* | | *6,540* | 654 | *100* | 0.003 | 0.0005 |
| *Escherichia coli* O157:H7 | *73,480* | *3,674* | 2,725 | 500 | *85* | 0.295 | 0.0083 |
| *E. coli*, non-O157 STEC | *36,740* | *1,837* | | | *85* | 0.295 | 0.0083 |
| *E. coli*, enterotoxigenic | *79,420* | | *2,090* | 209 | *70* | 0.005 | 0.0001 |
| *E. coli*, other diarrheogenic | *79,420* | | *2,090* | | 30 | 0.005 | 0.0001 |
| *Listeria monocytogenes* | *2,518* | *1,259* | 373 | | *99* | 0.922 | 0.2000 |
| *Salmonella* Typhi[b] | *824* | | 412 | | *80* | 0.750 | 0.0040 |
| *Salmonella*, nontyphoidal | *1,412,498* | *37,171* | 37,842 | 3,640 | *95* | 0.221 | 0.0078 |
| *Shigella* spp. | *448,240* | *22,412* | 17,324 | 1,476 | *20* | 0.139 | 0.0016 |
| Staphylococcus food poisoning | *185,060* | | *4,870* | 487 | *100* | 0.180 | 0.0002 |
| Streptococcus, foodborne | *50,920* | | *1,340* | 134 | *100* | 0.133 | 0.0000 |
| *Vibrio cholerae*, toxigenic | *54* | | 27 | | *90* | 0.340 | 0.0060 |
| *V. vulnificus* | *94* | | 47 | | *50* | 0.910 | 0.3900 |
| *Vibrio*, other | *7,880* | *393* | 112 | | *65* | 0.126 | 0.0250 |
| *Yersinia enterocolitica* | *96,368* | *2,536* | | | *90* | 0.242 | 0.0005 |
| Subtotal | *5,204,934* | | | | | | |
| **Parasitic** | | | | | | | |
| *Cryptosporidium parvum* | 300,000 | *6,630* | 2,788 | | *10* | 0.150 | 0.005 |
| *Cyclospora cayetanensis* | *16,264* | *428* | 98 | | *90* | 0.020 | 0.0005 |
| *Giardia lamblia* | *2,000,000* | *107,000* | 22,907 | | *10* | n/a | n/a |
| *Toxoplasma gondii* | *225,000* | | 15,000 | | *50* | n/a | n/a |
| *Trichinella spiralis* | *52* | | 26 | | 100 | 0.081 | 0.003 |
| Subtotal | *2,541,316* | | | | | | |
| **Viral** | | | | | | | |
| Norwalk-like viruses | *23,000,000* | | | | *40* | n/a | n/a |
| Rotavirus | *3,900,000* | | | | *1* | n/a | n/a |
| Astrovirus | *3,900,000* | | | | *1* | n/a | n/a |
| Hepatitis A | *83,391* | | 27,797 | | *5* | 0.130 | 0.0030 |
| Subtotal | *30,883,391* | | | | | | |
| **Grand Total** | ***38,629,641*** | | | | | | |

[a]Numbers in italics are estimates; others are measured.
[b]>70% of cases acquired abroad.

million illnesses each year, including 5.2 million (13%) due to bacteria, 2.5 million (7%) due to parasites, and 30.9 million (80%) due to viruses (Table 2). Overall, foodborne transmission accounts for 13.8 million of the 38.6 million illnesses (Table 3). Excluding illness caused by *Listeria*, *Toxoplasma*, and hepatitis A virus (three pathogens that typically cause nongastrointestinal illness), 38.3 million cases of acute gastroenteritis are caused by known pathogens, and 13.6 million (36%) of these are attributable to foodborne transmission. Among all illnesses attributable to foodborne transmission, 30% are caused by bacteria, 3% by parasites, and 67% by viruses.

### Hospitalizations

To estimate the number of hospitalizations due to foodborne transmission, we calculated for each pathogen the expected number of hospitalizations among reported cases by

Table 3. Estimated illnesses, hospitalizations, and deaths caused by known foodborne pathogens, United States

| | Illnesses | | | Hospitalizations | | | Deaths | | |
|---|---|---|---|---|---|---|---|---|---|
| Disease or agent | Total | Food-borne | % of total foodborne | Total | Food-borne | % of total foodborne | Total | Food-borne | % of total foodborne |
| **Bacterial** | | | | | | | | | |
| *Bacillus cereus* | 27,360 | 27,360 | 0.2 | 8 | 8 | 0.0 | 0 | 0 | 0.0 |
| Botulism, foodborne | 58 | 58 | 0.0 | 46 | 46 | 0.1 | 4 | 4 | 0.2 |
| *Brucella* spp. | 1,554 | 777 | 0.0 | 122 | 61 | 0.1 | 11 | 6 | 0.3 |
| *Campylobacter* spp. | 2,453,926 | 1,963,141 | 14.2 | 13,174 | 10,539 | 17.3 | 124 | 99 | 5.5 |
| *Clostridium perfringens* | 248,520 | 248,520 | 1.8 | 41 | 41 | 0.1 | 7 | 7 | 0.4 |
| *Escherichia coli* O157:H7 | 73,480 | 62,458 | 0.5 | 2,168 | 1,843 | 3.0 | 61 | 52 | 2.9 |
| *E. coli*, non-O157 STEC | 36,740 | 31,229 | 0.2 | 1,084 | 921 | 1.5 | 30 | 26 | 1.4 |
| *E. coli*, enterotoxigenic | 79,420 | 55,594 | 0.4 | 21 | 15 | 0.0 | 0 | 0 | 0.0 |
| *E. coli*, other diarrheogenic | 79,420 | 23,826 | 0.2 | 21 | 6 | 0.0 | 0 | 0 | 0.0 |
| *Listeria monocytogenes* | 2,518 | 2,493 | 0.0 | 2,322 | 2,298 | 3.8 | 504 | 499 | 27.6 |
| *Salmonella* typhi | 824 | 659 | 0.0 | 618 | 494 | 0.8 | 3 | 3 | 0.1 |
| *Salmonella*, nontyphoidal | 1,412,498 | 1,341,873 | 9.7 | 16,430 | 15,608 | 25.6 | 582 | 553 | 30.6 |
| *Shigella* spp. | 448,240 | 89,648 | 0.6 | 6,231 | 1,246 | 2.0 | 70 | 14 | 0.8 |
| Staphylococcus food poisoning | 185,060 | 185,060 | 1.3 | 1,753 | 1,753 | 2.9 | 2 | 2 | 0.1 |
| Streptococcus, foodborne | 50,920 | 50,920 | 0.4 | 358 | 358 | 0.6 | 0 | 0 | 0.0 |
| *Vibrio cholerae*, toxigenic | 54 | 49 | 0.0 | 18 | 17 | 0.0 | 0 | 0 | 0.0 |
| *V. vulnificus* | 94 | 47 | 0.0 | 86 | 43 | 0.1 | 37 | 18 | 1.0 |
| *Vibrio*, other | 7,880 | 5,122 | 0.0 | 99 | 65 | 0.1 | 20 | 13 | 0.7 |
| *Yersinia enterocolitica* | 96,368 | 86,731 | 0.6 | 1,228 | 1,105 | 1.8 | 3 | 2 | 0.1 |
| Subtotal | 5,204,934 | 4,175,565 | 30.2 | 45,826 | 36,466 | 59.9 | 1,458 | 1,297 | 71.7 |
| **Parasitic** | | | | | | | | | |
| *Cryptosporidium parvum* | 300,000 | 30,000 | 0.2 | 1,989 | 199 | 0.3 | 66 | 7 | 0.4 |
| *Cyclospora cayetanensis* | 16,264 | 14,638 | 0.1 | 17 | 15 | 0.0 | 0 | 0 | 0.0 |
| *Giardia lamblia* | 2,000,000 | 200,000 | 1.4 | 5,000 | 500 | 0.8 | 10 | 1 | 0.1 |
| *Toxoplasma gondii* | 225,000 | 112,500 | 0.8 | 5,000 | 2,500 | 4.1 | 750 | 375 | 20.7 |
| *Trichinella spiralis* | 52 | 52 | 0.0 | 4 | 4 | 0.0 | 0 | 0 | 0.0 |
| Subtotal | 2,541,316 | 357,190 | 2.6 | 12,010 | 3,219 | 5.3 | 827 | 383 | 21.2 |
| **Viral** | | | | | | | | | |
| Norwalk-like viruses | 23,000,000 | 9,200,000 | 66.6 | 50,000 | 20,000 | 32.9 | 310 | 124 | 6.9 |
| Rotavirus | 3,900,000 | 39,000 | 0.3 | 50,000 | 500 | 0.8 | 30 | 0 | 0.0 |
| Astrovirus | 3,900,000 | 39,000 | 0.3 | 12,500 | 125 | 0.2 | 10 | 0 | 0.0 |
| Hepatitis A | 83,391 | 4,170 | 0.0 | 10,841 | 90 | 0.9 | 83 | 4 | 0.2 |
| Subtotal | 30,833,391 | 9,282,170 | 67.2 | 123,341 | 21,167 | 34.8 | 433 | 129 | 7.1 |
| **Grand Total** | 38,629,641 | 13,814,924 | 100.0 | 181,177 | 60,854 | 100.0 | 2,718 | 1,809 | 100.0 |

multiplying the number of reported cases by pathogen-specific hospitalization rates from FoodNet data (23, 24), reported outbreaks (10, 25), or other published studies (Appendix). Not all illnesses resulting in hospitalization are diagnosed or reported. Health-care providers may not order the necessary diagnostic tests, patients may have already taken antibiotics that interfere with diagnostic testing, or the condition leading to hospitalization may be a sequela that develops well after resolution of the actual infection (e.g., *Campylobacter*-associated Guillain-Barré syndrome). Therefore, to account for underreporting, we doubled the number of hospitalizations among reported cases to derive for each pathogen an estimate of the total number of hospitalizations. Finally, we multiplied this figure by the proportion of infections attributable to foodborne transmission. Because of gaps in the available data, this approach could not be used for some parasitic and viral diseases (Appendix).

Overall, the pathogens listed in Table 2 cause an estimated 181,177 hospitalizations each year, of which 60,854 are attributable to foodborne transmission (Table 3). Excluding hospitalizations for infection with *Listeria*, *Toxoplasma*, and hepatitis A virus, 163,015 hospitalizations for acute gastroenteritis are caused by known pathogens, of which 55,512 (34%) are attributable to foodborne transmission. Overall, bacterial pathogens account for 60% of hospitalizations attributable to foodborne transmission, parasites for 5%, and viruses for 34%.

### Deaths

Like illnesses and hospitalizations, deaths are also underreported. Precise information on food-related deaths is especially difficult to obtain because pathogen-specific surveillance systems rarely collect information on illness outcome, and outcome-specific surveillance systems (e.g., death certificates) grossly underreport many pathogen-specific conditions. To estimate the number of deaths due to bacterial pathogens, we used the same approach described for hospitalizations: first calculating the number of deaths among reported cases, then doubling this figure to account for unreported deaths, and finally multiplying by the percentage of infections attributable to foodborne transmission. As with hospitalization, this approach could not be used for some parasitic and viral diseases.

Overall, the specified pathogens cause an estimated 2,718 deaths each year, of which 1,809 are attributable to foodborne transmission (Table 3). Excluding death due *to Listeria*, *Toxoplasma*, and hepatitis A virus, the number of deaths due to pathogens that cause acute gastroenteritis is 1,381, of which 931 (67%) are attributable to foodborne transmission. Bacteria account for 72% of deaths associated with foodborne transmission, parasites for 21%, and viruses for 7%. Five pathogens account for over 90% of estimated food-related deaths: *Salmonella* (31%), *Listeria* (28%), *Toxoplasma* (21%), Norwalk-like viruses (7%), *Campylobacter* (5%), and *E. coli* O157:H7 (3%).

### Food-Related Illness and Death from Unknown Pathogens

Some proportion of gastrointestinal illness is caused by foodborne agents not yet identified. This conclusion is supported by well-documented foodborne outbreaks of distinctive illness for which the causative agent remains unknown (e.g., Brainerd diarrhea) (26), by the large percentage of foodborne outbreaks reported to CDC for which no pathogen is identified (25), and by the large number of new foodborne pathogens identified in recent years.

To estimate food-related illness and death from unknown pathogens, we used symptom-based data to estimate the total number of acute gastrointestinal illnesses and then subtracted from this total the number of cases accounted for by known pathogens; this difference represents the illness due to acute gastroenteritis of unknown etiology. To determine how much of this illness was due to foodborne transmission, we used the percentages of foodborne transmission as determined above for acute gastroenteritis caused by known pathogens.

### Total Cases

To determine the rate of acute gastroenteritis in the general population, we used data on the frequency of diarrhea from the 1996 to 1997 FoodNet population survey. This survey did not collect data on the rate of vomiting among persons without diarrhea, however, so we relied on the Tecumseh and Cleveland studies for information on the frequency of this symptom. Because young children were overrepresented in the Tecumseh and Cleveland studies relative to the current U.S. population, rates of illness for these studies were age-adjusted. For the Tecumseh data, we used the reported age- and symptom-specific rates. For the Cleveland study, we used the method described by Garthright (27) to derive an overall age-adjusted rate of gastrointestinal illness; we then multiplied this rate by the relative frequency of symptoms to derive age-adjusted rates for specific symptoms.

In the 1996-97 FoodNet population survey, the overall rate of diarrhea was 1.4 episodes per person per year, and the rate of diarrheal illness, defined as diarrhea (3 loose stools per 24-hour period) lasting >1 day or interfering with normal activities, was 0.75 episodes per person per year (H. Herikstad, manuscript in preparation). We used the lower 0.75 rate for our analysis. To this we added the average age-adjusted rate of vomiting without diarrhea from the Tecumseh and Cleveland studies (0.30, Table 4) to derive an overall estimate of 1.05 episodes per person per year of acute gastrointestinal illness characterized by diarrhea, vomiting, or both.

Previous studies have shown that some cases of acute gastrointestinal illness are accompanied by respiratory symptoms; although the causes of these illnesses are generally unknown, such cases have traditionally been attributed to respiratory pathogens (20,27). Data on the frequency of concomitant respiratory symptoms were not collected in the 1996-97 FoodNet survey but were 20% to 27% among patients with acute gastroenteritis in the Tecumseh and Cleveland studies. Therefore, we adjusted downward our estimate of acute gastroenteritis by 25%, yielding a final estimate of 0.79 (1.05 X 0.75)

Table 4. Frequency of gastrointestinal illness in the general population, in episodes per person per year, as determined by three studies

| | FoodNet Population Survey | Tecumseh Study | | Cleveland Study | |
|---|---|---|---|---|---|
| Symptom | Age adjusted | Crude | Age adjusted | Crude | Age adjusted |
| Diarrhea or vomiting | -- | 0.98 | 0.81 | 1.28 | 0.87 |
| Diarrhea, any | 0.75 | 0.63 | 0.52 | 0.83 | 0.56 |
| Without vomiting | 0.61 | 0.40 | 0.33 | 0.48 | 0.33 |
| With vomiting | 0.14 | 0.23 | 0.19 | 0.35 | 0.23 |
| Vomiting without diarrhea | -- | 0.35 | 0.29 | 0.45 | 0.31 |

episodes of acute gastroenteritis per person per year. Extrapolated to a population of 267.7 million persons, the U.S. resident population in 1997 (28), this rate is equivalent to 211 million episodes each year in the United States.

As determined previously, 38.3 million of these 211 million episodes of acute gastroenteritis are attributable to known pathogens. A small proportion of the remaining 173 million episodes can be accounted for by known, noninfectious agents (e.g., mycotoxins, marine biotoxins); however, most are attributable to unknown agents. Because we cannot directly ascertain how many of these illnesses of unknown etiology are due to foodborne transmission, we used the relative frequency of foodborne transmission for known pathogens as a guide. For illnesses of known etiology, foodborne transmission accounts for 36% of total cases. Applying this percentage yields an estimate of 62 million cases of acute gastroenteritis of unknown etiology (36% of 173 million) due to foodborne transmission each year.

### Hospitalizations

The National Ambulatory Medical Care Survey/the National Hospital Ambulatory Medical Care Survey data were searched for visits due to symptoms of diarrhea, vomiting, or gastrointestinal infection (reason for visit classification {RVC} codes 1595, 1530, 1540) (17) and for visits resulting in a diagnosis of infectious enteritis (ICD-9-CM codes 001-009.3; Table 1). Visits associated with respiratory symptoms (RVC codes 1400-1499) or a diagnosis of influenza (ICD-9-CM code 487) were excluded. Data for the years 1992 to 1996 were combined before analysis. Overall, these criteria yielded an average of 15,810,905 visits annually from 1992 through 1996, of which an average of 1,246,763, or 7.9%, resulted in hospitalization. This figure is equivalent to a rate of 4.7 hospitalizations per 1,000 person-years.

The National Hospital Discharge Survey data were searched by using diagnostic codes for infectious gastroenteritis of known cause (ICD-9-CM codes 001-008; Table 1), with the exception of the code for *Clostridium difficile* colitis (ICD9 008.45), a common form of nosocomially acquired diarrhea. In addition, we included the nonspecific ICD-9-CM diagnosis codes 009 (infectious gastroenteritis) and 558.9 (other and unspecified noninfectious gastroenteritis and colitis). Despite the description, many of the illnesses attributed to ICD-9-CM code 558.9 are likely to be either infectious or due to agents possibly transmitted by food. For example, in the absence of laboratory testing, sporadic cases of viral gastroenteritis may be coded as 558.9. Under the previous ICD-8 classification, these same cases would have been assumed to be infectious and coded as 009 (29, 30). Data for the years 1992 to 1996 were weighted according to National Center for Health Statistics criteria and averaged to derive national estimates of annual hospitalizations. Records with a diagnosis of respiratory illness were not excluded because of the high incidence of respiratory infections among hospitalized patients.

Considering all listed diagnoses, the National Hospital Discharge Survey data for the years 1992 to 1996 yielded an annual average of 616,337 hospital discharges with a diagnosis of gastrointestinal illness. Included in this figure are 193,084 cases of gastroenteritis with an identified pathogen and an additional 423,293 cases of gastroenteritis of unknown etiology (Table 5). Converted to a rate, the total number is equivalent to 2.3 hospitalizations per 1,000 person-years. Because these data depend on the recording of a diagnosis and not just a symptom, it is likely that they underestimate the rate of hospitalization for acute gastroenteritis. This view is supported by FoodNet population survey data indicating a rate of approximately 7.2 hospitalizations per 1,000 person-years for

Table 5. Average annual hospitalizations and deaths for gastrointestinal illness by diagnostic category, National Hospital Discharge Survey, 1992–1996

| | 1st diagnosis | | All diagnoses | |
|---|---|---|---|---|
| Cause of enteritis[a] | Hospitalizations | Deaths | Hospitalizations | Deaths |
| Bacterial (001-005, 008-008.5) | 27,987 | 148[b] | 54,953 | 1,139 |
| Viral (008.6-008.8) | 82,149 | 0[b] | 132,332 | 194[b] |
| Parasitic (006-007) | 2,806 | 82[b] | 5,799 | 127[b] |
| Unknown etiology (009, 558.9) | 186,537 | 868[b] | 423,293 | 5,148 |
| Total | 299,479 | 1,898 | 616,377 | 6,608 |

[a]ICD-9-CM code.
[b]Estimate unreliable due to small sample size.

diarrheal illness (H. Herikstad, manuscript in preparation). These data were not included here because they omit hospitalizations for vomiting alone and are not easily adjusted for concomitant respiratory symptoms. Averaging the rates from the National Ambulatory Medical Care Survey/ National Hospital Ambulatory Medical Care Survey and National Hospital Discharge Survey yields a final estimate of 3.5 hospitalizations per 1,000 person-years, equivalent to 936,726 hospitalizations annually for acute gastroenteritis. As noted previously, 163,153 of these hospitalizations can be attributed to known causes of acute gastroenteritis, yielding an estimated 773,573 hospitalizations for acute gastroenteritis caused by unknown agents. Applying the relative frequency of foodborne transmission as determined for known pathogens yields an estimated 263,015 hospitalizations (34% of 773,573) for acute gastroenteritis due to foodborne transmission of unknown agents.

### Deaths

Multiple-cause-of-death data (16) and information on in-hospital-death data (National Hospital Discharge Survey) were used. ICD-9-CM codes 001-008 were employed to identify deaths due to diagnosed infectious gastroenteritis and ICD-9-CM codes 009 and 558 to identify deaths due to gastroenteritis of unknown etiology.

Death certificate data for the years 1992 to 1996 yielded an annual average of 6,195 total deaths, of which 1,432 (23%) were due to specific causes of gastroenteritis and 4,763 (77%) to undiagnosed causes of gastroenteritis. For the same years and ICD-9-CM codes, the average annual in-hospital deaths for all-listed diagnoses totaled 6,608, of which 1,460 were due to specific and 5,148 (77%) undiagnosed causes of gastroenteritis (Table 5). Averaging the totals for all causes from death certificate and National Hospital Discharge Survey data and adjusting to the 1997 U.S. census estimates, we estimated that gastroenteritis contributed to the death of 6,402 persons in the United States in 1997.

A total of 1,386 of these deaths can be explained by known causes of acute gastroenteritis (see above). Thus an estimated 5,016 deaths from acute gastroenteritis are caused by unknown agents. Applying the relative frequency of foodborne transmission as determined for known pathogens yields an estimated 3,360 deaths (67% of 5,016) due to acute gastroenteritis caused by foodborne transmission of unknown agents.

### Overall Food-Related Illness and Death

We summed illness attributable to foodborne gastroenteritis caused by known and unknown pathogens, yielding an estimate of 76 million illnesses, 318,574 hospitalizations, and 4,316 deaths. Adding to these figures the nongastrointestinal illness caused by *Listeria*, *Toxoplasma*, and hepatitis A virus, we arrived at a final national estimate of 76 million illnesses, 323,914 hospitalizations, and 5,194 deaths each year (Figure 1).

### Conclusions

The nature of food and foodborne illness has changed dramatically in the United States over the last century. While technological advances such as pasteurization and proper canning have all but eliminated some disease, new causes of foodborne illness have been identified. Researchers have used various methods to estimate the illnesses and deaths due to foodborne diseases in the United States. In 1985, Archer and Kvenberg coupled information on underreporting of salmonellosis with data on other foodborne pathogens to derive estimates of 8.9 million illnesses due to known pathogens and 24 million to 81 million illnesses due to all foodborne agents (2). In 1987, Bennett et al. computed incidence

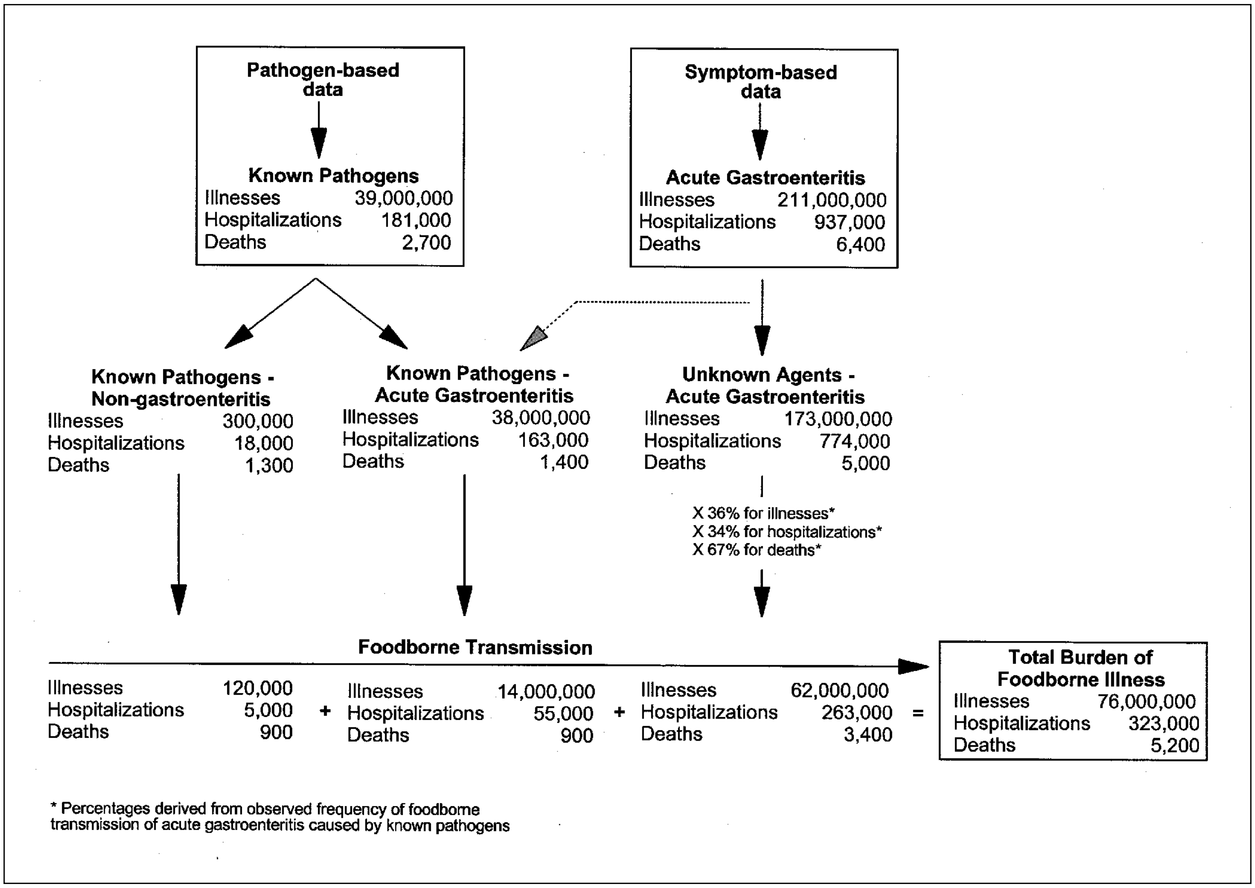

Figure. Estimated frequency of foodborne illness in the United States.

figures for all known infectious diseases and determined the proportion of each due to various modes of transmission. Summing these figures, they concluded that foodborne transmission of known pathogens caused 6.5 million illnesses and up to 9,000 deaths each year (3). In 1989, Todd used a combination of methods, including extrapolation from Canadian surveillance data, to derive an estimate of 12.5 million foodborne illnesses and 522 related deaths each year (4). Finally, in 1994, a task force convened by the Council for Agricultural Science and Technology (CAST) reviewed available studies and estimated the overall number of food-related illnesses at 33 million cases per year (5). These various estimates often refer to different entities. The estimates of 6.5 million and 8.9 million refer to illness caused by known pathogens, whereas the estimate of 33 million refers to all causes of foodborne illnesses, known and unknown, infectious and noninfectious.

Our estimates are based on data from a wide variety of sources and differ from previous estimates in several respects. For known pathogens, our estimate of 13.8 million illnesses per year is substantially higher than the previous estimates of 6.5 million and 8.9 million (2, 3), an increase attributable largely to our inclusion of foodborne illness caused by Norwalk-like viruses. For foodborne illness of all etiologies, our estimate of 76 million illnesses is within the range proposed by Archer and Kvenberg (2) but considerably higher than the point estimate of 33 million presented in the CAST report (5). Both our estimate and the CAST estimate assume that foodborne transmission accounts for ~35% of acute gastroenteritis cases caused by unknown agents. The disparity between the two stems from differences in the estimated annual frequency of acute gastroenteritis overall: 211 million cases for our estimate, 99 million for the CAST estimate.

Whereas our estimates of illness are generally higher than those of previous studies, our estimates of death are generally lower. We estimate that foodborne illness causes 5,020

deaths annually (1,810 deaths due to known pathogens and 3,210 deaths due to unknown agents), a total that is slightly more than half the 9,000 deaths estimated by Bennett et al. (3). The Bennett estimate includes 2,100 deaths due to campylobacteriosis, 1,200 deaths due to staphylococcal food poisoning, and 1,000 deaths due to trichinosis: our total for all three of these diseases is 101 deaths. Our estimated case-fatality rates for several other diseases are also lower than those used in the Bennett report, either because better data are available or perhaps because treatment has improved.

Our analysis suggests that unknown agents account for approximately 81% of foodborne illnesses and hospitalizations and 64% of deaths. Among cases of foodborne illness due to known agents, Norwalk-like viruses account for over 67% of all cases, 33% of hospitalizations, and 7% of deaths. The assumptions underlying the Norwalk-like viruses figures are among the most difficult to verify, and these percentages should be interpreted with caution (Appendix). Other important causes of severe illness are *Salmonella* and *Campylobacter*, accounting for 26% and 17% of hospitalizations, respectively. The leading causes of death are *Salmonella*, *Listeria,* and *Toxoplasma*, which together account for 1,427, or more than 75% of foodborne deaths caused by known pathogens. Many of the deaths due to toxoplasmosis occur in HIV-infected patients; recent advances in HIV treatment may greatly reduce deaths due to toxoplasmosis.

Of necessity, our analysis entails a number of assumptions. The first major assumption concerns the degree of underreporting. Well-documented estimates of underreporting are not available for most pathogens; therefore, we relied on multipliers derived for salmonellosis and other diseases. For salmonellosis, the multiplier of 38 has been independently derived by investigators in the United States using different data sources. The U.S. figure is five to tenfold higher than multipliers for *Salmonella* and *Campylobacter* recently derived in Great Britain (31). However, this difference is nearly or wholly offset by far higher per capita rates of reported infections in Great Britain. Nevertheless, when extrapolated to other pathogens, these multipliers may result in under- or overestimates, and clearly studies such as those conducted for *Salmonella* are needed to develop better multipliers for these other diseases. However, in our analysis, changing the multipliers for individual diseases has a minimal effect on the overall estimate of foodborne illness.

Our second set of assumptions concerns the frequency of foodborne transmission for individual pathogens. We have used published studies when available, but these are rare. As with underreporting multipliers, errors affect estimates for individual pathogens but have minimal effect on the estimate of overall illness and death from foodborne diseases. The one notable exception is the estimate for Norwalk-like viruses. Because these viruses account for an especially large number of illnesses, changes in the percentage attributed to foodborne transmission have a major effect on our overall estimates. For example, if the actual number of infections due to foodborne transmission were 30% rather than 40%, the overall estimate would decrease from 76 million to 63 million illnesses per year. Interestingly, our overall estimate is influenced far less by the Norwalk-like virus case estimate itself. It would require a 100-fold reduction in the estimated number of Norwalk-like virus cases to reduce the overall estimate from 76 million to 63 million.

A third assumption concerns the frequency of acute gastroenteritis in the general population. The rate we used is based in part on recent data from the FoodNet population survey, a retrospective survey involving more than 9,000 households. The overall rate of diarrhea as recorded by the survey was 1.4 episodes per person per year; however, we used the survey's far lower rate of 0.75 episodes of diarrheal illness per person per year. Furthermore, we limited our definition of acute gastroenteritis to symptoms of diarrhea or vomiting and reduced the rate to account for concomitant respiratory symptoms. As a result, our final assumed rate of 0.79 episodes of acute gastroenteritis per person per year is very similar to respiratory-adjusted estimates derived from the prospectively conducted Tecumseh (0.74) and Cleveland (0.71) studies (27). All three studies are based on household surveys, and thus the rates of illness are not influenced by changes in health-care delivery. Compared with rates of diarrheal illness from studies conducted in Great Britain, our estimated rate is higher than in one recent study (31) but lower than another (32).

In addition to these assumptions, our analysis has several limitations. Differences in available surveillance information prevented us from using the same method to estimate illness and death from bacterial, parasitic, and viral pathogens. Furthermore, because of a paucity of surveillance information, we did not include specific estimates for some known, occasionally foodborne pathogens (e.g., *Plesiomonas, Aeromonas, or Edwardsiella)*, nor did we develop specific estimates for known noninfectious agents, such as mushroom or marine biotoxins, metals, and other inorganic toxins. However, many of these agents cause gastroenteritis and are therefore captured in our overall estimate of foodborne illness. With the exception of a few important pathogens (Appendix), we have not estimated the number of cases of chronic sequelae, although these may be part of the overall burden of foodborne diseases. Finally, future research will refine our assumptions and allow for more precise estimates.

Methodologic differences between our analysis and previously published studies make it difficult to draw firm conclusions regarding overall trends in the incidence of foodborne illness. In general, the differences between our estimates and previously published figures appear to be due primarily to the availability of better information and new analyses rather than real changes in disease frequency over time. For example, *E. coli* O157:H7 was estimated to cause 10,000 to 20,000 illnesses annually, based on studies of patients visiting a physician for diarrhea. Recent FoodNet data have allowed a more detailed estimation of mild illnesses not resulting in physician consultation. Our estimate of nearly 74,000 illnesses per year incorporates these milder illnesses and should not be misconstrued as demonstrating a recent increase in *E. coli* O157:H7 infections. Whatever the limitations on retrospective comparisons, the estimates presented here provide a more reliable benchmark with which to judge the effectiveness of ongoing and future prevention efforts.

Further refinements of foodborne disease estimates will require continued and improved active surveillance. Beginning in 1998, the FoodNet population survey was modified to capture cases of vomiting not associated with diarrhea; further enhancement to capture concomitant respiratory symptoms should refine the FoodNet survey data. Expansion of laboratory diagnostic capacity could lead to better detection of certain pathogens, estimates of the degree of underreporting for additional diseases, and estimates of the proportion of specific diseases transmitted through food. Heightened surveillance for acute, noninfectious foodborne diseases, such as mushroom poisoning and other illnesses caused by biotoxins, could further improve estimates of illness and death from foodborne illness. Emergency department-based surveillance systems (33) or poison control center-based surveillance might provide such information. Finally, identifying new causes of enteric illness and defining the public health importance of known agents (e.g., enteroaggregative *E. coli*) would improve foodborne disease prevention efforts.

### Appendix

Methods, assumptions, and references for pathogen-specific estimates

### Bacterial Pathogens

**Pathogen:** *Bacillus cereus*

**Reported cases:** Cases not routinely reported. Because it is a mild illness, reported cases assumed to be 10 times the average annual number of outbreak-related cases reported to CDC, 1983-1992 (10,25).

**Total cases:** Assumed to be 38 times the number of reported cases by extrapolation from studies of salmonellosis.

**Hospitalization rate:** Determined from outbreaks reported to CDC, 1982-1992 (10,25) and (CDC, unpub. data).

**Case-fatality rates:** Determined from outbreaks reported to CDC, 1982-1992 (10,25), including those associated with nursing homes (34).

**Percent foodborne:** Although infection occasionally occurs through other routes, case estimates presented are based on foodborne outbreaks and are therefore assumed to reflect only foodborne transmission.

**Pathogen:** *Clostridium botulinum*

**Reported cases:** Average annual number of cases of foodborne botulism reported to CDC, 1992-1997 (7).

**Total cases:** Because it is a severe illness, assumed to be two times the number of reported cases.

**Hospitalization rate:** Determined from outbreaks reported to CDC, 1982-1992 (10,25) and (CDC, unpub. data).

**Case-fatality rate:** Based on outbreaks reported to CDC, 1982-1992 (10,25).

**Percent foodborne:** 100% by definition.

**Pathogen:** *Brucella* spp.

**Reported cases:** Average annual number of cases reported to CDC, 1992-1997 (7).

**Total cases:** Assumed to be 14 times reported cases, based on published estimates that 4% to 10% of cases are reported (35).

**Hospitalization rate:** Determined from outbreaks reported to CDC, 1982-1992 (10,25) and (CDC, unpub. data).

**Case-fatality rate:** Historically 2% to 5% (36).

**Percent foodborne:** Overall, consumption of milk or cheese products from Mexico implicated in 45% of cases reported from California from 1973 to 1992 (37). Because the proportion of cases due to foodborne transmission was higher in the latter half of this period, we assumed that currently 50% of cases are foodborne.
**Comments:** Reports from California or Texas account for most of cases in recent years.

**Pathogen:** *Campylobacter* spp.
**Reported cases:** Outbreak-related cases based on reports to CDC, 1983-1992 (10,25). Passive surveillance estimate based on average number of cases reported to CDC, 1992-1994 (CDC, unpub. data). Active surveillance estimate based on extrapolation of average 1996-1997 FoodNet rate (24.1 cases per 100,000 population) to 1997 U.S. population (23).
**Total cases:** Assumed to be 38 times the number of reported cases, based on studies of salmonellosis. Resulting estimate is roughly comparable with midpoint rate estimate from Tauxe (38) for *C. jejuni* (1,020 cases per 100,000 population), applied to 1997 population. Assumes minimal contribution from non-jejuni *Campylobacter*.
**Hospitalization rate:** Based on hospitalization rate for culture-confirmed cases reported to FoodNet, 1996-1997 (23,24).
**Case-fatality rate:** Based on case-fatality rate for culture-confirmed cases reported to FoodNet, 1996-1997 (23,24).
**Percent foodborne:** Although waterborne outbreaks occur, foodborne transmission accounts for most of the sporadic cases (38).
**Comments:** Guillain-Barré syndrome (GBS) is an acute flaccid paralysis that can occur several weeks after infection with various agents, including *Campylobacter*. The incidence of GBS has been estimated at 1.7 cases per 100,000 population, and serologic studies suggest that ~30% of patients with GBS have evidence of recent infection with *Campylobacter* (39). Based on these figures, we estimate that ~1,360 cases of *Campylobacter*-associated GBS occurred in the United States in 1997.

**Pathogen:** *Clostridium perfringens*
**Reported cases:** Cases not routinely reported. Because it is a mild illness, number of reported cases assumed to be 10 times the average annual number of outbreak-related cases reported to CDC, 1983-1992 (10,25).
**Total cases:** Assumed to be 38 times the number of reported cases, by extrapolation from studies of salmonellosis.
**Hospitalization rate:** Determined from outbreaks reported to CDC, 1982-1992 (10,25) and (CDC, unpub. data).
**Case-fatality rate:** Based on reported outbreaks, 1983-1992 (10,25).
**Percent foodborne:** 100% (40). Case estimates presented are based on foodborne outbreaks and therefore reflect foodborne transmission of *C. perfringens*, type A.

**Pathogen:** *Escherichia coli* O157:H7
**Reported cases:** Passive surveillance estimate based on average number of cases reported to CDC through the National Electronic Telecommunications System for Surveillance (NETSS), 1995-1998; data from the Public Health Laboratory Information System (PHLIS) were used for those states not reporting to NETSS during this time period (7). Passive surveillance data for 1998 are provisional. Active surveillance estimate based on an extrapolation of a weighted average of the FoodNet rate for the years 1996-1997 to the 1997 U.S. population (23,24). A weighted average was used because the overall FoodNet rate is disproportionately influenced by a high rate in a single northern state with a relatively small population. Because the incidence of infection is thought to be generally higher in northern states (41), we weighted the crude rate derived from FoodNet by the total population of each participating state. The weighted rate (1.34 cases per 100,000 population) was used when extrapolating the FoodNet rate to the total U.S. population.
**Total cases:** Studies conducted in FoodNet sites suggest that 13-27 cases of *E. coli* O157:H7 infection occur in the community for each confirmed case that is reported (22). To estimate total cases, we multiplied the number of reported cases, as determined through active surveillance, by 20, the midpoint of this estimate.
**Hospitalization rate:** Based on the hospitalization rate for culture-confirmed cases reported to FoodNet, 1996-1997 (23,24).
**Case-fatality rate:** Case-fatality rate based on mortality associated with sporadic cases reported to FoodNet, 1996-1997 (23,24).
**Percent foodborne:** Based on outbreaks of known source reported to CDC, 1982-1997 (CDC, unpub. data). Person-to-person transmission assumed to be secondary to foodborne transmission (2).
**Comments:** Our estimate of total cases is considerably higher than previous estimates based on patients seeking care for diarrhea. Our estimate includes patients with far milder illness and should not be interpreted as indicating an increase in incidence. Hemolytic uremic syndrome (HUS) occurs in ~4% of all reported cases. Based on our estimate of total cases and active surveillance cases, between 2,954 and 147 patients are expected to contract HUS each year.

**Pathogen:** *E. coli*, Shiga toxin-producing serogroups other than O157 (STEC)
**Reported cases:** Cases not routinely reported; many clinical laboratories cannot identify.
**Total cases:** Assumed to be half as common as infection with *E. coli* O157:H7. Early studies suggest that the incidence of non-O157 STEC infections is 20%-30% that of *E. coli* O157:H7 in North America (42, 43); however, more recent studies using different techniques suggest that this figure should be 50% (44,45).
**Hospitalization rate:** Assumed to be comparable with *E. coli* O157:H7, but may be lower (46).
**Case-fatality rate:** Assumed to be comparable with *E. coli* O157:H7, but may be lower (46).
**Percent foodborne:** Assumed to be comparable with *E. coli* O157:H7.
**Comment:** Although non-O157 STEC can cause hemolytic uremic syndrome, the relative frequency of this complication is unknown. Reports from Canada suggest that non-O157 STEC are the cause of at least 7% (47) and possibly as many as 20% (48) of HUS cases.

**Pathogen:** *E. coli,* enterotoxigenic
**Reported cases:** Not routinely reported. Outbreak-related cases based on average for 18 outbreaks reported to CDC from 1975 through 1997 (CDC, unpub. data). Reported cases assumed to be 10 times the number of outbreak-related cases.
**Total cases**: Assumed to be 38 times the number of reported cases by extrapolation from studies of salmonellosis.
**Hospitalization rate:** Low; assumed to be 0.5% of cases.
**Case-fatality rate:** Serious illness is generally restricted to infants in developing countries. Based on experience with reported outbreaks, assumed to be 1 in 10,000 cases in the United States.
**Percent foodborne:** Nearly all outbreaks reported to CDC from 1975 through 1997 have been foodborne (CDC, unpub. data); many sporadic cases are associated with travel to other countries where both water and foodborne exposures are likely.

**Pathogen:** *E. coli,* other diarrheogenic
**Reported cases:** Not routinely reported. Assumed to be at least as common as enterotoxigenic *E. coli* (ETEC) based on limited information from studies in North America and Europe (49).
**Total cases:** Assumed equal to ETEC.
**Hospitalization rate:** Assumed equal to ETEC.
**Case-fatality rate:** Assumed equal to ETEC.
**Percent foodborne:** Very little data available. As few foodborne outbreaks have been reported, it is assumed that only 30% of cases are foodborne.
**Comment:** This category includes enteropathogenic, enteroaggregative, and enteroinvasive *E. coli*, as well as poorly defined pathogenic groups (50). Although little is known about the incidence of these infections in the United States, these pathogens have been linked to both outbreaks and sporadic illnesses. Limited studies suggest that the importance of some of these organisms in the United States is seriously underestimated (see Nataro and Kaper [49]). Although clearly a heterogeneous collection of organisms, we assume that these pathogens as a group have similar modes of transmission and mortality rates as ETEC.

**Pathogen:** *Listeria monocytogenes*
**Reported cases:** Rates from FoodNet, 1996-1997, (23,24) and comparable sentinel site surveillance (51), extrapolated to the 1997 U.S. population.
**Total cases:** Because it is a severe illness, assumed to be 2 times the number of reported cases.
**Hospitalization rate:** Based on hospitalization rate for culture-confirmed cases reported to FoodNet, 1996-1997 (23,24).
**Case-fatality rate:** Based on published reports (51), 1996-1997 FoodNet data (23,24), and recent outbreaks (CDC, unpub. data).
**Percent foodborne:** Although foodborne transmission accounts for all reported domestic outbreaks (52), the potential for nosocomial transmission has been demonstrated (53).
**Comments:** Figures include both perinatal and nonperinatal disease. FoodNet data on hospitalization indicate that nearly 90% of reported cases result in hospitalization (24).

**Pathogen:** *Salmonella* Typhi
**Reported cases:** Average number of cases reported to CDC, 1992-1997 (7).
**Total cases:** Because it is a severe illness, assumed to be two times the number of reported cases.
**Hospitalization rate:** Rate of hospitalization based on published outbreak reports (54,55).
**Case-fatality rate:** Based on outcomes of 2,254 cases reviewed by Mermin (56).
**Percent foodborne:** Although waterborne outbreaks have been reported in the United States, foodborne transmission is believed to account for most cases (3).
**Comments:** Over 70% percent of reported cases are associated with foreign travel (56).

**A. Pathogen:** *Salmonella*, nontyphoidal
**B. Reported cases:** Outbreak-related cases based on reports to CDC, 1983-1992 (10,25). Passive surveillance estimate based on average number of cases reported to CDC, 1992-1997 (57). Active surveillance estimate based on extrapolation of the average 1996-1997 FoodNet rate to the 1997 U.S. population (23).
**Total cases:** Assumed to be 38 times the number of reported cases based on FoodNet data (Voetsch, manuscript in preparation) and the "sequential surveillance artifact" multiplier derived by Chalker and Blaser (21).
**Hospitalization rate:** Based on hospitalization rate for culture-confirmed cases reported to FoodNet, 1996-1997 (23,24).
**Case-fatality rate:** Average case-fatality rate among cases reported to FoodNet, 1996-1997 (23,24). This rate is lower than the previously published rate of 1.3% (58).
**Percent foodborne:** Although occasionally associated with exposure to pets, reptiles, and contaminated water, salmonellosis is primarily a foodborne disease (59).

**Pathogen:** *Shigella* spp.
**Reported cases:** Outbreak-related cases based on reports to CDC, 1983-1992 (10,25). Passive surveillance estimate based on average number of cases reported annually to CDC, 1992-1997 (57). Active surveillance estimate based on extrapolation of average 1996-1997 FoodNet rate to the 1997 U.S. population (23).
**Total cases:** Because *Shigella* frequently causes bloody diarrhea, total cases assumed to be 20 times the number of reported cases, based on similarity to *E. coli* O157:H7.
**Hospitalization rate:** Based on hospitalization rate for culture-confirmed cases reported to FoodNet, 1996-1997 (23,24).
**Case-fatality rate:** Average case-fatality rate among cases reported to FoodNet, 1996-1997 (23,24).
**Percent foodborne:** Assumed to be 20%. Although most cases are due to person-to-person transmission (60), foodborne outbreaks are responsible for a substantial number of cases (61).

**Pathogen:** *Staphylococcus aureus* (enterotoxin)
**Reported cases:** Not routinely reported. Assumed to be 10 times the number of foodborne outbreak-related cases reported to CDC, 1983-1992 (10,25).
**Total cases**: Assumed to be 38 times the number of reported cases, by extrapolation from studies of salmonellosis.
**Hospitalization rate:** Determined from outbreaks reported to CDC, 1982-1992 (10,25), (CDC, unpub. data), and published reports (62).
**Case-fatality rate:** Determined from reported outbreaks to CDC, 1977-1992 (10,25,63).
**Percent foodborne:** 100% by definition. Case estimates presented are based on foodborne outbreaks and therefore reflect foodborne transmission.
**Comment:** The number of outbreak-associated cases of staphylococcal food poisoning reported to CDC has decreased substantially since 1973 (Bean and Griffin, 1990). This decrease is unlikely to be an artifact of decreased recognition: there has been no compensatory increase in the number of foodborne outbreaks of unknown etiology with an incubation period consistent with staphylococcal food poisoning (CDC, unpub. data).

**Pathogen:** *Streptococcus,* Group A
**Reported cases:** Not routinely reported. Assumed to be 10 times the number of foodborne outbreak-related cases reported to CDC, 1982-1992 (10,25).
**Total cases:** Assumed to be 38 times the number of reported cases, by extrapolation from studies of salmonellosis.
**Hospitalization rate:** Determined from outbreaks reported to CDC, 1982-1992 (10,25) and CDC, unpub. data.
**Case-fatality rate:** Determined from outbreaks reported to CDC, 1982-1992 (10).
**Percent foodborne:** 100% foodborne by definition. Case estimates presented are based on foodborne outbreaks and therefore reflect foodborne transmission.

**Pathogen:** *Vibrio cholerae*, toxigenic O1 or O139
**Reported cases:** Based on cases reported to CDC, 1988-1997 (7).
**Total cases:** Assumed that the number of clinically significant illnesses is two times the number of reported cases.
**Hospitalization rate:** Based on cases reported to CDC, 1992-1994 (64).
**Case-fatality rate:** Based on cases reported to CDC, 1992-1994 (64).
**Percent foodborne:** Assumed to be primarily foodborne. Most reported cases linked to foodborne outbreaks, and at least 65% of sporadic cases may be foodborne (64).
**Comments:** 96% of cases acquired abroad (64).

**Pathogen:** *Vibrio vulnificus*
**Reported cases:** Cases reported to CDC from 22 states, 1988-1996 (65).
**Total cases:** Because it is a severe illness, assumed to be two times the number of reported cases.
**Hospitalization rate:** Based on overall rate among cases reported to CDC, 1988-1996 (65).
**Case-fatality rate:** Based on overall rate among cases reported to CDC, 1988-1996; death rate higher among cases due to foodborne transmission (65).
**Percent foodborne:** Based on Shapiro et al. (65).
**Comment:** Most cases are reported by Gulf States (Florida, Alabama, Louisiana, Texas).

**Pathogen:** *Vibrio,* other spp.
**Reported cases:** Passive surveillance estimate based on cases reported to CDC, 1988-1996 (CDC, unpub. data). Active surveillance estimate based on 1996 FoodNet rate extrapolated to the 1997 U.S. population (23). FoodNet data from 1997 not included because of a large outbreak of *Vibrio parahaemolyticus* infections that could falsely elevate the overall rate.
**Total cases:** Because it is a moderately severe illness, total cases assumed to equal 20 times the reported cases, a degree of underreporting comparable with *E. coli* O157:H7 infections.
**Hospitalization rate:** Based on rate among non-*vulnificus*, non-*cholerae* O1 cases reported by Hlady (66).
**Case-fatality rate:** Based on rate among non-*vulnificus*, non-*cholerae* O1 cases reported by Hlady (66).
**Percent foodborne:** Based on history of shellfish consumption for cases reported by Hlady (66).
**Comment:** Because of larger sample size, data from Hlady (66) used in preference to FoodNet data for hospitalization and death rates.

**Pathogen:** *Yersinia enterocolitica*
**Reported cases:** Active surveillance estimate based on extrapolation of average 1996-1997 FoodNet rate to the 1997 U.S. population (23,24).
**Total cases:** Assumed to be 38 times the number of reported cases, based on studies of salmonellosis.
**Hospitalization rate:** Based on the hospitalization rate for culture-confirmed cases reported to FoodNet, 1996-1997 (23,24).
**Case-fatality rate:** Low, assumed to be 0.5% (23).
**Percent foodborne:** Assumed to be 90%. Nearly all reported outbreaks in United States have been linked to contaminated foods, and pork is specifically believed to be the source of most infections (67).

### Parasitic Pathogens

**Pathogen:** *Cryptosporidium parvum*
**Reported cases:** Passive surveillance estimate based on the average annual number of cases reported to CDC, 1995-1997 (7). Active surveillance estimate based on extrapolation of the average 1997-98 FoodNet rate to the 1997 U.S. population (6,24).
**Total cases**: Published studies suggest that ~2% of all stools tested for *Cryptosporidium* are positive (68, 69). We assume this rate of infection applies to all patients visiting a health-care provider for acute gastroenteritis. Using an estimate of ~15 million physician visits for diarrhea each year (see text), we estimate there are approximately 300,000 cases of cryptosporidiosis per year. This figure is 45-fold higher than the estimated number of reported cases based on FoodNet active surveillance, a multiplier only slightly larger than the one used for salmonellosis.
**Hospitalization rate:** Based on the hospitalization rate for culture-confirmed cases reported to FoodNet, 1997-1998 (6,24).
**Case-fatality rate:** Average case-fatality rate among cases reported to FoodNet, 1997-1998 (6,24).
**Percent foodborne:** Based on very limited information (70-72), we assume that 10% of cases are attributable to foodborne transmission, with the rest due to consumption of contaminated water or person-to-person transmission.
**Comment:** Cryptosporidiosis in AIDS is associated with a severe protracted course of diarrhea (73).

**Pathogen:** *Cyclospora cayetanensis*
**Reported cases:** Passive surveillance estimate based on average annual number of cases reported to CDC, 1995-1997 (7). Active surveillance estimate based on extrapolation of average 1997-1998 FoodNet rate to the 1997 U.S. population (6,24).
**Total cases:** Assumed to be 38 times the number of reported cases based on studies of salmonellosis.
**Hospitalization rate:** Based on the hospitalization rate for culture-confirmed cases reported to FoodNet, 1997 (24).
**Case-fatality rate:** Very low (74,75). Assumed to be 0.05%, comparable with *Clostridium perfringens*.
**Percent foodborne:** Assumed 90% foodborne, based on recent reported outbreaks (74,75).

**Pathogen:** *Giardia lamblia*
**Reported cases:** Not routinely reported.
**Total cases:** Sensitive surveillance in two sites (Vermont and Wisconsin) suggests a rate of 40 cases per 100,000 persons per year (76,77). In addition, an estimated 5% of all cases are reported. Thus, approximately 100,000 cases will be detected each year, representing 2,000,000 actual cases.
**Hospitalization rate:** An estimated 5,000 cases per year are severe enough to require hospitalization.
**Case-fatality rate:** Exceedingly low. Assumed to be no more than 10 deaths annually.
**Percent foodborne:** Assumed to be 10%. Recreational water is probably the major source of transmission (76-78); however, several foodborne outbreaks have been reported (79,80).

**Pathogen:** *Toxoplasma gondii*
**Reported cases:** Not routinely reported.

**Total cases:** Based on national serologic data collected during the 1994 NHANES, approximately 40% of persons ≥60 years old are seropositive for toxoplasmosis (CDC, unpub. data). Assuming equal rates of infection over time, at least 0.6% of the population experiences an acute infection each year, representing approximately 1,500,000 infections per year. Approximately 15% of infections are symptomatic.
**Hospitalization rate:** Varies widely according to host immune status. Data from NHDS indicate that from 1992 to 1996, toxoplasmosis was the first listed diagnosis for approximately 5,000 hospital discharges each year. We have used this figure as a conservative estimate of the number of actual hospitalizations.
**Case-fatality rate:** Varies widely according to host immune status. Of the approximately 5,000 hospital discharges annually for which toxoplasmosis is the first listed diagnosis, approximately 750 involve a deceased patient. We have used this figure as a conservative estimate of the number of actual deaths.
**Percent foodborne:** Although the proportion associated with eating contaminated food varies by geographic region, we assume an overall average of 50%. Recent unpublished data from Europe suggest that 60% of acute infections are from contaminated food (Ruth Gilbert, pers. comm.).
**Comment**: Typically, infection with *Toxoplasma gondii* produces an asymptomatic illness or a mild viral-like febrile illness with lymphadenopathy. Acute diarrhea is not commonly associated with acute infection. Estimates from the Massachusetts Department of Health suggest that one case of congenital toxoplasmosis occurs for every 10,000 births (81). Extrapolating to 4,000,000 live births in the United States, an estimated 400 children are born with congenital toxoplasmosis. Based on calculations by investigators from Stanford University, each year approximately 6,000 women who experience an acute infection during pregnancy and who do not receive treatment give birth to a child with congenital toxoplasmosis, which results in chronic sequelae (82). During an outbreak of toxoplasmosis in British Columbia, of an estimated 2,900-7,700 infections, 19 cases of retinitis were reported. If there are at least 150,000 symptomatic cases annually, from 300 to 1,050 cases (0.2% to 0.7%, respectively) of ocular toxoplasmosis could occur. If there are 300,000 cases, from 600 to 2,100 ocular cases could occur. Thus, there could be from 300 to 2,100 ocular cases of toxoplasmosis annually. An estimated 4,000 persons with AIDS develop *Toxoplasma* encephalitis annually. In summary, from (400+300+4,000) = 4,700 to (6,000+2,100+4,000) = 12,100 persons develop chronic sequelae due to toxoplasmosis each year.

**Pathogen:** *Trichinella spiralis*
**Reported cases:** Based on NETSS surveillance data, approximately 40 cases are reported annually.
**Total cases:** Because it can be a severe illness, assumed to be two times the number of reported cases.
**Hospitalization rate:** Based on outbreak-related cases reported to CDC, 1982-1992 (10).
**Case-fatality rate:** Assumed to be 0.3% based on data from a large series in Europe.
**Percent foodborne:** 100% (83)
**Comment:** Clinically, acute trichinosis may be asymptomatic or may have acute gastrointestinal symptoms, followed by a parenteral phase of fever and myalgias. In 10% to 20% of cases neurologic or cardiac symptoms develop, many severe and potentially leading to chronic illness.

### Viral Pathogens

**Pathogen:** Rotavirus
**Reported cases:** Not routinely reported.
**Total cases:** Because every child has at least one symptomatic infection (84-86), the number of cases is assumed to equal the 1997 U.S. birth cohort (3.9 million).
**Hospitalizations**: 50,000 (87,88).
**Case-fatality rate:** Very low: 20 to 40 deaths per year (89).
**Percent foodborne**: probably very low (<1%) (90).

**Pathogen:** Astrovirus
**Reported cases:** Not routinely reported.
**Total cases:** Because every child has at least one symptomatic infection, the number of cases is assumed to equal the 1997 US birth cohort (3.9 million).
**Hospitalizations**: Assumed to equal 25% of number of hospitalizations for rotavirus (= 12,500) (91).
**Case-fatality rate:** Very low (<10 deaths per year).
**Percent foodborne:** Probably very low (<1%) (91).

**Pathogen**: Norwalk-like viruses (NLV).
**Reported cases:** Not routinely reported.
**Total cases:** Very few data are available for assessing the disease burden associated with Norwalk-like viruses, and very few studies have been conducted using the most sensitive diagnostics for NLVs. One community-based study from the Netherlands found 17% of cases of acute gastroenteritis were associated with Norwalk-like viruses, compared with 6% of controls, using reverse transcriptase polymerase chain reaction (RT-PCR) for detection of NLVs (92). An Australian study detected NLVs in 15% of hospitalized patients using immune electron microscopy (93). Studies have generally been conducted exclusively among young children or used less sensitive detection methods (electron microscopy); in these studies, NLVs have been detected in ~1% to 5% of participants (94-98). However, a recent study incorporating RT-PCR for viral detection among children 2 months to 2 years of age found that 21% of cases of acute gastroenteritis were associated with NLVs (99). Given these data, we assume that 11% of all episodes of acute primary gastroenteritis are due to NLVs (using the data from the best of the studies) (92).
**Hospitalizations**: NLV assumed to account for 11% of 452,000 annual hospitalizations for viral gastroenteritis (100).
**Case-fatality rate:** Low. NLV assumed to account for 11% of an estimated 2,800 fatal cases of viral gastroenteritis each year (100).
**Percent foodborne:** We assume that the proportion of all NLV-associated illness that is foodborne is 40%. This estimate is based on a recent report which found that 47% of NLV-associated acute gastroenteritis outbreaks in the United States in which the modes of transmission were known were foodborne (101). Since we would assume that foodborne-associated outbreaks might be more likley to be reported than Norwalk-like virus-associated outbreaks with other mechanisms of spread, the proportion was lowered to 40%. This estimate is in general agreement with other reviews (102-104). No data are available to directly determine the proportion of cases of NLV-associated disease attributable to foodborne transmission.

**Pathogen**: Hepatitis A
**Reported cases:** Based on cases reported to CDC, 1992-1997 (7).

**Total cases:** Assumed to be three times the number of reported cases (105).
**Hospitalizations**: Thirteen percent; based on data from CDC Sentinel Counties Studies (106);
**Case-fatality rate:** 0.3%; based on data from the viral Hepatitis Surveillance Program and the CDC Sentinel Counties Studies (105,107). Deaths calculated by applying the case-fatality rate to reported cases.
**Percent foodborne**: Foodborne transmission accounts for approximately 5% of outbreaks of known source (105). Note that the source is not determined in approximately 50% of hepatitis A outbreaks, and foodborne transmission could account for a far higher percentage of cases.

### Acknowledgments

We thank Fred Angulo, Beth Bell, Thomas Breuer, Cindy Friedman, Roger Glass, Eric Mintz, Steven Ostroff, Morris Potter, David Swerdlow, Tom Van Gilder, and two anonymous reviewers for their comments.

Dr. Mead is a medical epidemiologist with the Foodborne and Diarrheal Diseases Branch, CDC, in Atlanta, Georgia. His professional interests include infectious diseases surveillance, outbreak investigations, and interventions to prevent foodborne illness.

### References

1. Bryan FL. Diseases transmitted by foods. Atlanta: Centers for Disease Control; 1982.
2. Archer DL, Kvenberg JE. Incidence and cost of foodborne diarrheal disease in the United States. J Food Protect 1985;48:887-94.
3. Bennett J, Holmberg S, Rogers M, Solomon S. Infectious and parasitic diseases. In: Amler R, Dull H, editors. Closing the gap: the burden of unnecessary illness. New York: Oxford Univ Press; 1987: 102-14.
4. Todd ECD. Preliminary estimates of costs of foodborne disease in the United States. J Food Protect 1989;52:595-601.
5. Foodborne pathogens: risks and consequences. Ames, IA: Council of Agricultural Science and Technology; 1994.
6. 1998 FoodNet Surveillance Results. Preliminary Report. Atlanta: Centers for Disease Control and Prevention; 1999.
7. Summary of notifiable diseases, United States, 1997. MMWR Morb Mortal Wkly Rep 1997;46(54).
8. Bean NH, Martin SM, Bradford H. PHLIS: an electronic system for reporting public health data from remote sites. Am J Public Health 1992;82:1273-76.
9. Levine W, Griffin P, Gulf Coast Vibrio Working Group. Vibrio infections on the Gulf Coast: results of first year regional surveillance. J Infect Dis 1993;167:479-83.
10. Foodborne disease outbreaks, 5-year summary, 1983-1987. MMWR 1992;39 (SS-1):15-57.
11. Woodwell DA. National Ambulatory Medical Care Survey: 1996 Summary. Advance data from vital and health statistics; no. 295. Hyattsville, Maryland: National Center for Health Statistics; 1997.
12. McCaig LF, McLemore T. Plan and operation of the National Hospital Ambulatory Medical Care Survey. Hyattsville: National Center for Health Statistics; 1994.
13. McCaig LF. National Hospital Ambulatory Medical Care Survey: 1996 Outpatient Department Summary. Advance data from vital and health statistics: no. 294. Hyattsville, Maryland: National Center for Health Statistics; 1997.
14. McCaig LF, Stussman BJ. National Hospital Ambulatory Medical Care Survey: 1996 Emergency Department Summary. Advance data from vital and health statistics: no. 293. Hyattsville, Maryland: National Center for Health Statistics; 1997.
15. Graves EJ, Gillium BS. Detailed diagnoses and procedures, National Hospital Discharge Survey, 1995. National Center for Health Statistics. Vital Health Stat 1997;13.
16. NCHS. Public use data tape documentation. Multiple cause of death for ICD-9. Hyattsville, Maryland: Public Health Service; 1998.
17. Schneider D, Appleton L, McLemore T. A reason for visit classification for ambulatory care. Hyattsville, Maryland: National Center for Health Statistics; 1979.
18. Public Health Service and Health Care Financing Administration. International classification of diseases, 9th Revision, Clinical Modification. Washington D.C.: Public Health Service; 1991.
19. Monto AS, Koopman JS. The Tecumseh Study. XI. Occurrence of acute enteric illness in the community. Am J Epidemiol 1980;112:323-333.
20. Dingle JH, Badger GF, Jordan W. Gastrointestinal illness. In: Illness in the home. A study of 25,000 illness in a group of Cleveland families. Cleveland: The Press of Western Reserve University; 1964: 129-61.
21. Chalker R, Blaser M. A review of human salmonellosis: III. Magnitude of *Salmonella* infection in the United States. Rev Infect Dis 1988;10:111-24.
22. Hedberg C, Angulo F, Townes J, Vugia D, Farley M, FoodNet. Differences in *Escherichia coli* O157:H7 annual incidence among FoodNet active surveillance sites. Baltimore, MD; 1997 June 22-26, 1997.
23. 1996 Final FoodNet surveillance report. Atlanta: Centers for Disease Control and Prevention; 1998.
24. 1997 Final FoodNet surveillance report. Atlanta: Centers for Disease Control and Prevention; 1998.
25. Surveillance for foodborne-disease outbreaks—United States, 1988-1992. MMWR 1996;45(No. SS-5):2-55.
26. Parsonnet J, Wanke CA, Hack H. Idiopathic chronic diarrhea. In: Blaser MJ, Smith PD, Ravdin JI, Greenberg HB, Guerrant RL, editors. Infections of the gastrointestinal tract. New York: Raven Press, Ltd; 1995: 311-23.
27. Garthright WE, Archer DL, Kvenberg JE. Estimates of incidence and cost of infectious diseases in the United States. Pub Health Reports 1988;103:107-15.
28. Population estimates. Available at <http://www.census.gov/population/www/estimates/popest.html> ed: Bureau of the Census, Economics and Statistics Administration, US Department of Commerce.
29. Helmick CG, Griffin PM, Addiss DG, Tauxe RV, Juranek DD. Infectious diarrheas. In: Everhart JE, editor. Digestive diseases in the United States: epidemiology and impact. U.S. Department of Health and Human Service, National Institutes of Health, National Institute of Diabetes and Digestive Diseases. Washington, D.C.: U.S. Government Printing Office; 1994: 85-123.

30. Dugger BC, Lewis WF. Comparability of diagnostic data coded by the 8th and 9th revisions of the international classification of diseases. Washington, D.C.: U.S. Government Printing Office 1987: DHHS publication no. (PHS) 87-1378. (Vital and health statistics: series 2, no. 104).
31. Wheeler JG, Sethi D, Cowden JM, Wall PG, Rodrigues LC, Tompkins DS, et al. Study of infectious intestinal disease in England: rates in the community, presenting to general practice, and reported to national surveillance. The Infectious Intestinal Disease Study Executive. BMJ 1999;318:1046-50.
32. Feldman RA, Banatvala N. The frequency of culturing stools from adults with diarrhea in Great Britain. Epidemiol Infect 1994;113:41-4.
33. Talan DA, Moran GJ, Mower WR, Newdow M, Ong S, Slutsker L, et al. Emergency ID NET: an emergency department-based emerging infections sentinel network. Ann Emerg Med 1998;32:703-11.
34. Levine W, Smart J, Archer D, Bean N, Tauxe R. Foodborne disease outbreaks in nursing homes, 1975 through 1987. JAMA 1991;266:2105-09.
35. Taylor JP, Perdue JN. The changing epidemiology of human brucellosis in Texas. Am J Epidemiol 1989;130:160-5.
36. Dalrymple-Champneys W. Brucella infection and undulant fever in man. London: Oxford University Press; 1960.
37. Chomel B, DeBess E, Mangiamele D, Reilly K, Farver T, Sun R, et al. Changing trends in the epidemiology of human brucellosis in California from 1973 to 1992: a shift toward foodborne transmission. J Infect Dis 1994;170:1216-23.
38. Tauxe R. Epidemiology of *Campylobacter jejuni* infections in the United States and other industrialized nations. In: Nachamkin, Blaser M, Tompkins L, editors. *Campylobacter jejuni*: current status and future trends; 1992. p. 9-19.
39. Mishu B, Blaser MJ. Role of infection due to *Campylobacter jejuni* in the initiation of Guillain-Barré syndrome. Clin Infec Dis 1993;17:104-8.
40. Bartlett JG. Gas gangrene (other *Clostridium*-associated diseases). In: Mandell GL, Douglas RG, Bennett JE, editors. Principles and practice of infectious diseases. Third ed. New York: Churchill Livingstone; 1990: 1850-60.
41. Slutsker L, Ries AA, Greene KD, Wells JG, Hutwagner L, Griffin PM. *Escherichia coli* O157:H7 diarrhea in the United States: clinical and epidemiologic features. Ann Intern Med 1997;126:505-13.
42. Pai CH, Ahmed N, Lior H, Johnson WM, Sims HV, Woods DE. Epidemiology of sporadic diarrhea due to verocytotoxin-producing *Escherichia coli*: a two-year prospective study. J Infect Dis 1988;157:1054-7.
43. Bokete TN, O'Callahan CM, Clausen CR, Tang NM, Tran N, Moseley SL, et al. Shiga-like toxin-producing *Escherichia coli* in Seattle children: a prospective study. Gastroenterology 1993;105:1724-31.
44. Acheson DWK, Breuker SD, Donohue-Rolfe A, Kozak K, Yi A, Keusch GT. Development of a clinically useful diagnostic enzyme immunoassay for enterohemorrhagic *Escherichia coli* infection. In: Karmali MA, Goglio AG, editors. Recent advances in verocytotoxin-producing *Escherichia coli* infections. Amsterdam: Elsevier Science B. V.; 1994: 109-12.
45. Park CH, Gates KM, Vandel NM, Hixon DL. Isolation of Shiga-like toxin producing *Escherichia coli* (O157 and non-O157) in a community hospital. Diagn Microbiol Infect Dis 1996;26:69-72.
46. Tarr PI, Neill MA. Perspective: the problem of non-O157:H7 shiga toxin (Verocytotoxin)-producing *Escherichia coli* [comment]. J Infect Dis 1996;174:1136-9.
47. Rowe PC, Orrbine E, Lior H, Wells GA, McLaine PN. A prospective study of exposure to verotoxin-producing *Escherichia coli* among Canadian children with haemolytic uraemic syndrome. The CPKDRC co-investigators. Epidemiol Infect 1993;110:1-7.
48. Johnson R, Clark R, Wilson J, Read S, Rhan K, Renwick S, et al. Growing concerns and recent outbreaks involving non-O157:H7 serotypes of verocytoxigenic *Escherichia coli*. J Food Protect 1996;59:1112-22.
49. Nataro JP, Kaper JB. Diarrheogenic *Escherichia coli*. Clin Microbiol Rev 1998;11:1-60.
50. Hedberg CW, Savarino SJ, Besser JM, Paulus CJ, Thelen VM, Myers LJ, et al. An outbreak of foodborne illness caused by *Escherichia coli* O39:NM, an agent not fitting into the existing scheme for classifying diarrheogenic *E. coli*. J Infect Dis 1997;176:1625-8.
51. Tappero J, Schuchat A, Deaver K, Mascola L, Wenger J. Reduction in the incidence of human listeriosis in the United States. Effectiveness of prevention efforts. JAMA 1995;273:1118-22.
52. Slutsker L, Schuchat A. Listeriosis in Humans. In: Ryser E, Marth E, editors. *Listeria*, listeriosis, and food safety. New York: Marcel Dekker; 1999: 75-96.
53. Schuchat A, Lizano C, Broome C, Swaminathan B, Kim C, Winn K. Outbreak of neonatal listeriosis associated with mineral oil. Pediatr Infect Dis J 1991;10:183-9.
54. Hoffman TA, Ruiz CJ, Counts GW, Sachs JM, Nitzkin JL. Waterborne typhoid fever in Dade County, Florida. Clinical and therapeutic evaluation of 105 bacteremic patients. Am J Med 1975;59:481-7.
55. Klotz SA, Jorgensen JH, Buckwold FJ, Craven PC. Typhoid fever. An epidemic with remarkably few clinical signs and symptoms. Arch Intern Med 1984;144:533-7.
56. Mermin J, Townes J, Gerber M, Dolan N, Mintz E, Tauxe R. Typhoid fever in the United States,1985-1994. Arch Intern Med 1998;158:633-638.
57. Laboratory confirmed Salmonella surveillance. Annual Summary, 1997. Atlanta, Georgia: Centers for Disease Control and Prevention; 1999.
58. Cohen M, Tauxe R. Drug-resistant *Salmonella* in the United States: an epidemiologic perspective. Science 1986;234:964-9.
59. Tauxe R. *Salmonella*: a postmodern pathogen. J Food Protect 1991;54:563-8.
60. DuPont HL. Shigella species. In: Mandell GL, Douglas RG, Bennett JE, editors. Principles and practice of infectious diseases. 3rd ed. New York: Churchill Livingstone; 1990: 1716-22.
61. Black RE, Craun GF, Blake PA. Epidemiology of common-source outbreaks of shigellosis in the United States, 1961-1975. Am J Epidemiol 1978;108:47-52.
62. Levine WC, Bennett RW, Choi Y, Henning KJ, Rager JR, Hendricks KA, et al. Staphylococcal food poisoning caused by imported canned mushrooms. J Infect Dis 1996;173:1263-7.

63. Holmberg S, Blake P. Staphylococcal food poisoning in the United States. JAMA 1984;251:487-9.
64. Mahon B, Mintz E, Greene K, Wells J, Tauxe R. Reported cholera in the United States, 1992-1994. JAMA 1996;276:307-12.
65. Shapiro RL, Altekruse S, Hutwagner L, Bishop R, Hammond R, Wilson S, et al. The role of Gulf Coast oysters harvested in warmer months in *Vibrio vulnificus* infections in the United States, 1988-1996. Vibrio Working Group. J Infect Dis 1998;178:752-9.
66. Hlady W, Klontz K. The epidemiology of *Vibrio* infections in Florida, 1981-1993. J Infect Dis 1996;173:1176-83.
67. Ostroff S. Yersinia as an emerging infection: epidemiologic aspects of Yersiniosis. Contributions to Microbiology & Immunology 1995;13:5-10.
68. Skeels MR, Sokolow R, Hubbard CV, Andrus JK, Baisch J. Cryptosporidium infection in Oregon public health clinic patients, 1985-88: the value of statewide laboratory surveillance. Am J Public Health 1990;80:305-8.
69. Roberts CL, Morin C, Addiss DG, Wahlquist SP, Mshar PA, Hadler JL. Factors influencing Cryptosporidium testing in Connecticut. J Clin Microbiol 1996;34:2292-3.
70. Petersen C. Cryptosporidium and the food supply. Lancet 1995;345:1128-9.
71. Djuretic T, Wall PG, Nichols G. General outbreaks of infectious intestinal disease associated with milk and dairy products in England and Wales: 1992 to 1996. Commun Dis Rep CDR Wkly 1997;7:R41-5.
72. Outbreaks of *Escherichia coli* O157:H7 infection and cryptosporidiosis associated with drinking unpasteurized apple cider--Connecticut and New York, October 1996. MMWR 1997;46:4-8.
73. Petersen C. Cryptosporidiosis in patients infected with the human immunodeficiency virus [see comments]. Clin Infect Dis 1992;15:903-9.
74. Herwaldt BL, Ackers ML. An outbreak in 1996 of cyclosporiasis associated with imported raspberries. The Cyclospora Working Group [see comments]. N Engl J Med 1997;336:1548-56.
75. Herwaldt BL, Beach MJ. The return of *Cyclospora* in 1997: another outbreak of cyclosporiasis in North America associated with imported raspberries. Cyclospora Working Group [see comments]. Ann Intern Med 1999;130:210-20.
76. Addiss DG, Davis JP, Roberts JM, Mast EE. Epidemiology of giardiasis in Wisconsin: increasing incidence of reported cases and unexplained seasonal trends. Am J Trop Med Hyg 1992;47:13-9.
77. Birkhead G, Vogt RL. Epidemiologic surveillance for endemic *Giardia lamblia* infection in Vermont. The roles of waterborne and person-to-person transmission. Am J Epidemiol 1989;129:762-8.
78. Dennis DT, Smith RP, Welch JJ, Chute CG, Anderson B, Herndon JL, et al. Endemic giardiasis in New Hampshire: a case-control study of environmental risks. J Infect Dis 1993;167:1391-5.
79. Petersen LR, Cartter ML, Hadler JL. A food-borne outbreak of *Giardia lamblia*. J Infect Dis 1988;157:846-848.
80. Osterholm MT, Forfang JC, Ristinen TL, Dean AG, Washburn JW, Godes JR, et al. An outbreak of foodborne giardiasis. N Engl J Med 1981;304:24-8.
81. Guerina NG, Hsu HW, Meissner HC, Maguire JH, Lynfield R, Stechenberg B, et al. Neonatal serologic screening and early treatment for congenital *Toxoplasma gondii* infection. The New England Regional Toxoplasma Working Group [see comments]. N Engl J Med 1994;330:1858-63.
82. Wong SY, Remington JS. Toxoplasmosis in pregnancy [see comments]. Clin Infect Dis 1994;18:853-61.
83. Capo V, Despommier DD. Clinical aspects of infection with *Trichinella* spp. Clinical Microbiology Reviews 1996;9:47-54.
84. Tucker A, Haddix A, Bresee J, Holman R, Parashar U, Glass R. Cost-effectiveness analysis of a rotavirus immunization program for the United States. JAMA 1998;279:1371-76.
85. Rodriguez WJ, Kim HW, Brandt CD, Schwartz RH, Gardner MK, Jeffries B, et al. Longitudinal study of rotavirus infection and gastroenteritis in families served by a pediatric medical practice: clinical and epidemiologic observations. Pediatr Infect Dis J 1987;6:170-6.
86. Gurwith M, Wenman W, Hinde D, Feltham S, Greenberg H. A prospective study of rotavirus infection in infants and young children. J Infect Dis 1981;144:218-24.
87. Parashar UD, Holman RC, Clarke MJ, Bresee JS, Glass RI. Hospitalizations associated with rotavirus diarrhea in the United States, 1993 through 1995: surveillance based on the new ICD-9-CM rotavirus-specific diagnostic code. J Infect Dis 1998;177:13-7.
88. Jin S, Kilgore PE, Holman RC, Clarke MJ, Gangarosa EJ, Glass RI. Trends in hospitalizations for diarrhea in United States children from 1979 through 1992: estimates of the morbidity associated with rotavirus. Pediatr Infect Dis J 1996;15:397-404.
89. Kilgore PE, Holman RC, Clarke MJ, Glass RI. Trends of diarrheal disease-associated mortality in US children, 1968 through 1991. JAMA 1995;274:1143-48.
90. Kapikian AZ, Chanock RM. Rotaviruses. In: Fields BN, DM DMK, Howley PM, et al, editors. Fields Virology. 3rd ed. Philadelphia: Lippincott-Raven; 1996: 1657-708.
91. Glass RI, Noel J, Mitchell D, Herrmann JE, Blacklow NR, Pickering LK, et al. The changing epidemiology of astrovirus-associated gastroenteritis: a review. Arch Virol - Suppl 1996;12:287-300.
92. Koopmans M, van Duynhoven Y, van de Heide R, et al. Molecular detection and epidemiology of Norwalk-like viruses and Sapporo-like viruses in the Netherlands. Presented at the International Workshop on Human Caliciviruses, Atlanta, Georgia, USA; 1999 Mar 29-31.
93. Grohman G. Viral diarrhoea in children in Australia. In: Tzipori S, et al, editors. Infectious diarrhoea of the young. New York: Elsevier Science Publishers; 1985.
94. Wolfaardt M, Taylor MB, Booysen HF, Englebrecht L, Grabow WOK, Jiang X. Incidence of human calicivirus and rotavirus infection in patients with gastroenteritis in South Africa. J Med Virol 1997;51:290-6.
95. Vial P, Kotloff KL, Tall BD, Morris JG, Levine MM. Detection by immune electron microscopy of 27-nm viral particles associated with community-acquired diarrhea in children. J Infect Dis 1989;161:571-3.
96. Donneli G, Ruggeri FM, Tinari A, Marziano ML, Menichella D, Caione D, et al. A three-year diagnostic and epidemiologic study on viral infantile diarrhoea in Rome. Epidemiol Infect 1988;100:311-20.

97. Riepenhoff-Talty M, Saif LJ, Barrett HJ, Suzuki H, Ogra PL. Potential spectrum of etiologic agents of viral enteritis in hospitalized patients. J Clin Microbiol 1983;17:352-6.
98. Suzuki H, Konno T, Kutsuzawa T, Imai A, Tazawa F, Ishida N, et al. The occurrence of calicivirus in infants with acute gastroenteritis. J Med Virol 1979;4:321-6.
99. Pang X, Joensuu J, Vesikari T. Human calicivirus-associated sporadic gastroenteritis in Finnish children less than 2 years of age followed prospectively during a rotavirus vaccine trial. Pediatr Infect Dis J 1999;18:420-6.
100. Mounts A, Holman R, Clarke M, Bresee J, Glass R. Trends in hospitalizations associated with gastroenteritis among adults in the United States, 1979-1995. Epi Infect . In press 1999.
101. Fankhauser RL NJ, Monroe SS, Ando T, Glass RI. Molecular epidemiology of "Norwalk-like viruses" in outbreaks of gastoenteritis in the United States. J Infect Dis 1998;178:1571-8.
102. Kaplan JE, Feldman R, Campbell DS, Lookabaugh C, Gary WG. The frequency of a Norwalk-like pattern of illness in outbreaks of acute gastroenteritis. Am J Pub Health 1982;72:1329-2.
103. Sekine S, Okada S, Hayashi Y. Prevalence of small round structured virus infections in acute gastroenteritis outbreaks in Tokyo. Microbiol Immunol 1989;33:207-17.
104. Viral Gastroenteritis Sub-Committee of the PHLS Virology Committee. Outbreaks of gastroenteritis associated with SRSVs. PHLS Microbiol Digest 1998;10:1-8.
105. Hepatitis surveillance report no. 56. Atlanta: U.S. Department of Health and Human Services, Public Health Service, CDC. 1996.
106. Bell BP, Shapiro CN, Alter MJ, Moyer LA, Judson FN, Mottram K, et al. The diverse patterns of hepatitis A epidemiology in the United States. Implications for vaccination strategies. J Infect Dis 1998;178:1579-84.
107. Hoofnagle JH, Carithers RL, Shapiro C, Ascher N. Fulminant hepatic failure. Summary of a workshop. Hepatology 1995:21:240-252.
